# Trends of Eye and Adnexa‐Related Hospital Admissions in Lebanon: An Epidemiological Retrospective Study

**DOI:** 10.1002/hsr2.72296

**Published:** 2026-04-06

**Authors:** Dany Akiki, Riwa Ibrahim, Mohammad Al Zein, Mona Bou Khdoud, Maria Maalouf, Jamal Bleik

**Affiliations:** ^1^ Department of Ophthalmology, Gilbert and Rose‐Marie Chagoury School of Medicine Lebanese American University Beirut Lebanon; ^2^ Faculty of Medical Sciences Lebanese University Beirut Lebanon

**Keywords:** admission rate, adnexa, epidemiological study, eye, hospitalization, lebanon, trends

## Abstract

**Background and Aim:**

Visual impairment represents a major public health concern, affecting millions of individuals worldwide. In recent years, the global burden of eye diseases and vision impairment has continued to grow. This study aims to investigate the trends in eye and adnexa‐related hospital admissions in Lebanon from 2006 to 2019, as covered by the Ministry of Public Health (MOPH). The primary objective is to assess trends by disease type and gender.

**Methods:**

This retrospective observational study was conducted in Lebanon from 2006 to 2019. Data on eye and adnexa‐related hospital admissions covered by the MOPH were collected with ethical clearance from the Institutional Review Board (IRB) of the Lebanese American University (LAU). The study utilized ICD‐10 codes to classify admissions and analyze gender‐specific rates of hospitalization.

**Results:**

Between 2006 and 2019, Lebanon recorded a total of 113,514 hospital admissions related to eye and adnexal diseases. Females accounted for 62,462 (55%) of these admissions, with an average of 4461 admissions per year, while males accounted for 51,052 (45%), averaging 3646 admissions annually. The primary causes for hospitalization were disorders of the lens, glaucoma, disorders of the eyelid, lacrimal system and orbit, and disorders of the vitreous body and globe. Over the study period, the overall hospital admission rate increased from 141.42 per 100,000 in 2006 to 149.78 per 100,000 in 2019 (*p*‐value < 0.001).

**Conclusion:**

There was an increasing trend in eye and adnexa‐related hospital admissions in Lebanon from 2006 to 2019, with notable gender disparities and shifts in the primary causes of hospitalization. These findings highlight the importance of continuous monitoring and intervention strategies to address the evolving healthcare needs related to eye and adnexal diseases in Lebanon and worldwide.

## 1 Introduction

Visual impairment is a significant public health challenge, affecting millions of individuals worldwide. According to the World Health Organization's (WHO) report issued in 2019, over 2.2 billion people worldwide experience a certain degree of visual impairment [1]. The majority of cases are attributable to age‐related conditions and refractive errors [2]. Visual impairment is associated with a substantial decline in quality of life [3], and certain underlying etiologies are linked to systemic diseases and increased mortality [4].

Refractive errors, such as myopia and presbyopia, remain the leading cause of vision impairment, with uncorrected refractive errors affecting approximately 123 million people worldwide, and uncorrected presbyopia affecting around 826 million individuals. Cataract, an age‐related process, affects about 65 million people globally [5]. Other major global causes of vision impairment include glaucoma, diabetic retinopathy, and age‐related macular degeneration [6].

Eye conditions that do not directly impair vision can be equally debilitating. These include disorders affecting the eye itself or the ocular adnexa, which include structures closely associated with the eye, such as the orbit, eyelids, eyelashes, lacrimal apparatus, and extraocular muscles. Conditions such as blepharitis, conjunctivitis, dry eye syndrome, and chalazion are often painful, sometimes disabling, and represent a common reason for seeking ophthalmic care worldwide [2].

Advanced age remains the primary risk factor for numerous ocular diseases. Genetic factors also play an established role in the pathophysiology of several age‐related eye conditions. Modifiable risk factors include lifestyle factors such as smoking, occupational exposure, and nutrition [5].

The global demand for eye care is expected to rise, driven primarily by population growth, population aging, and lifestyle changes [7]. Data regarding the cause‐specific prevalence of vision impairment are essential for public health policies, including resource allocation, service planning, and prioritization of scientific advances [8].

Previous studies in Australia and England and Wales have shown clear age and gender‐related patterns in hospital admissions for eye and adnexa disorders, with lens disorders being the most common cause in Australia and increasing rates linked to aging, lifestyle changes, and improved screening [2, 9]. To complete the global picture, we conducted a similar study in Lebanon to analyze national trends in eye and adnexa‐related hospital admissions from 2006 to 2019.

## 2 Methods

### 2.1 Study Design

This is an observational, retrospective, epidemiological study evaluating the trends of eye and adnexa‐related hospital admissions in Lebanon between 2006 and 2019. The study population includes patients residing in Lebanon who were admitted to Lebanese hospitals for ophthalmological concerns and covered by the MOPH. The primary outcome is the percentage and rate of hospital admissions, stratified by gender and disease type, based on the International Statistical Classification of Diseases and Related Health Problems 10th Revision (ICD‐10) codes applied to all admissions recorded during the study period, along with the percentage of change in hospital admission rates for each disease type.

The eye and adnexa codes, ranging from H00 to H59, cover a wide spectrum of disorders including eyelid, orbit, and lacrimal systems disorders (H00‐H05), conjunctival disorders (H10‐H11), corneal, scleral, ciliary body, and iris disorders (H15‐H22), lenticular disorders (H25‐H28), retinal and choroidal disorders (H30‐H36), glaucoma (H40‐H42), vitreous body and globe disorders (H43‐H44), optic nerve and visual pathway disorders (H46‐H47), ocular muscles, binocular movement, refraction, and accommodation disorders (H49‐H52), blindness and visual disturbances (H53‐H54), other eye and adnexal disorders (H55‐H57), and intra‐ and postoperative complications (H59‐H59) [10].

### 2.2 Ethical Clearance

Approval to proceed with this study was obtained after submitting an exempt review application to the Institutional Review Board (IRB) of the Lebanese American University (LAU). A data access agreement was also secured from the MOPH. Data were retrieved from the MOPH through the office of Information Technology (IT), and included hospital inpatient admissions, without patients' identifiers. Moreover, data were obtained from the statistical bulletin tables that are publicly accessible on the official website of the Lebanese MOPH [11].

### 2.3 Statistical Analysis

This study is reported in accordance with the STROBE and SAMPL guidelines for observational studies and statistical reporting [12, 13]. Microsoft Excel program was used to collect data. Data were analyzed using IBM Statistical Package for the Social Sciences (SPSS) for Windows, version 23.0 (IBM Corp., Armonk, NY, USA).

Hospital admission rates for diseases of the eye and adnexa were calculated per 100,000 population for each year from 2006 to 2019. Descriptive statistics were used to summarize frequencies, proportions, and annual rates. Ninety‐five percent confidence intervals (95% CI) were computed where appropriate. Data regarding the population size of Lebanon were collected from the latest world population prospect of the United Nations [14].

Temporal trends in admission rates were assessed using the chi‐square test for trend (Cochran‐Armitage test). This test was applied to evaluate changes over time in overall admission rates as well as in ICD‐10 category‐specific and sex‐stratified rates. These analyses were pre‐specified to examine nationwide temporal patterns of hospital admission for eye and adnexa diseases. Subgroup analyses by sex and by disease category were considered exploratory.

All statistical tests were two‐sided, and a *p*‐value < 0.05 was considered statistically significant. Statistical terms, abbreviations, and symbols (e.g., CI: confidence interval; χ²: chi‐square) are defined at first use.

### 2.4 Lebanese Health Care System

Lebanon's health system is based on a mixed public‐private model involving multiple key organizations. The MOPH plays a central regulatory role and acts as the insurer of last resort for uninsured citizens, while the National Social Security Fund (NSSF) provides health coverage for formally employed private‐sector workers. Additional public coverage is offered through the Civil Servants Cooperative (CSC) and the health funds of the military and security forces. Alongside these public payers, private health insurance companies play a significant role, particularly for higher‐income groups. Health service delivery is dominated by private hospitals and clinics, complemented by public hospitals, the primary health care network coordinated by the MOPH, and a wide range of non‐governmental organizations (NGOs) that contribute substantially to primary care, humanitarian assistance, and refugee health services [15]. In 2003, a unified Beneficiaries Database was created, including beneficiaries of the MOPH, NSSF, CSC, Army, and the Security Forces. The unification of beneficiaries' information implied that double‐coverage or double‐billing from more than one fund was no longer possible for beneficiaries, and thus considerably improved the efficiency of the system [16].

## 3 Results

Lebanon's resident population, excluding Palestinian camps and Syrian refugees, increased from 3,867,300 in 2006 to 4,546,618 in 2019 [11], distributed across its nine governorates (Beirut, Mount Lebanon, North Lebanon, South Lebanon, Beqaa, Nabatiyeh, Akkar, Baalbek‐Hermel, and Keserwan‐Jbeil). As of 2025, the Lebanese Order of Physicians (LOP) registers 457 practicing ophthalmologists nationwide, corresponding to approximately 8.32 ophthalmologists per 100,000 residents, higher than the global average of 5.68 per 100,000 [17, 18].

A total of 113,514 hospital admissions related to the eye and adnexa were recorded in Lebanon between 2006 and 2019. Females accounted for 62,462 (55%) of these admissions, averaging 4,461 admissions per year, while males accounted for 51,052 (45%), corresponding to 3,646 admissions per year. The most common cause of hospitalization was disorders of the lens, with 87,548 cases (77.3%), followed by glaucoma and disorders of the eyelid, lacrimal system, and orbit, with 3,965 cases (3.4%) (Table 1). The annual hospital admission rate for eye and adnexa‐related diseases increased from 141.42 per 100,000 in 2006 to 149.78 per 100,000 in 2019, representing an overall increase of 5.9% (*p* < 0.001) (Table 2).

**Table 1:** Percentage of hospital admissions from total number of admissions per ICD code during the study period.

ICD Code	Diseases of the Eye and Adnexa	Total Number	Percentage From Total Number of Admissions
H00–H06	Disorders of eyelid, lacrymal system, and orbit	3,855	3.4%
H10–H13	Disorders of conjuctiva	1,538	1.4%
H15–H22	Disorders of sclera, cornea, iris, and ciliary body	2,425	2.1%
H25–H28	Disorders of lens	87,548	77.3%
H30–H36	Disorders of choroid and retina	3,134	2.8%
H40–H42	Glaucoma	3,965	3.4%
H43–H45	Disorders of vitrous body and globe	3,375	3.0%
H46–H48	Disorders of optic nerve and visual pathways	147	0.1%
H49–H52	Disorders of ocular muscles, binocular movement accomodation and refraction	2,442	2.1%
H53–H54	Visual disturbances and blindness	2,429	2.2%
H55–H59	Other disorders of eye and adnexa	2,656	2.3%

**Table 2:** Percentage of Change in the Hospital Admission Rates for Diseases of the Eye and Adnexa.

Diseases of the eye and adnexa	Number of Diseases in 2006	Rate of Diseases in 2006 per 100,000 Persons (95% CI)	Number of Diseases in 2019	Rate of Diseases in 2019 per 100,000 Persons (95% CI)	Percentage of Change from 2006–2019	*p*‐value
Disorders of eyelid, lacrymal system and orbit	223	4.72 (4.11–5.33)	215	3.72 (3.23–4.21)	−21.3%	0.01+
Disorders of conjunctiva	77	1.63 (1.27–1.99)	146	2.53 (2.13–2.93)	+54.8%	0.002*
Disorders of sclera, cornea, iris, and ciliary body	189	4.00 (3.43–4.57)	104	1.80 (1.45–2.15)	−55.1%	< 0.001**
Disorders of lens	4,826	102.25 (99.46–105.04)	6,941	120.05 (117.33–122.77)	+17.4%	< 0.001**
Disorders of choroid and retina	207	4.39 (3.80–4.98)	379	6.55 (5.90–7.20)	+49.5%	< 0.001**
Glaucoma	424	8.98 (8.14–9.82)	126	2.18 (1.80–2.56)	−75.7%	< 0.001**
Disorders of vitrous body and globe	142	3.01 (2.53–3.49)	251	4.34 (3.81–4.87)	+44.3%	< 0.001**
Disorders of optic nerve and visual pathways	6	0.13 (0.03–0.23)	15	0.26 (0.13–0.39)	+104.1%	0.14
Disorders of ocular muscles, binocular movement accomodation and refraction	239	5.06 (4.42–5.70)	112	1.94 (1.58‐2.30)	−61.7%	< 0.001**
Visual disturbances and blindness	94	1.99 (1.59–2.39)	272	4.70 (4.15–5.25)	+136.2%	< 0.001**
Other disorders of eye and adnexa	248	5.25 (4.60–5.90)	99	1.71 (1.37–2.05)	−67.4%	< 0.001**
Total	6,675	141.42 (138.14–144.70)	8,660	149.78 (146.54–153.02)	+5.9%	< 0.001**

Abbreviation: CI, confidence interval.

^+^ Significant: *p*‐value ≤ 0.05;

* Very significant: *p*‐value ≤ 0.01;

** Highly significant: *p*‐value ≤ 0.001

**Figure 1:**
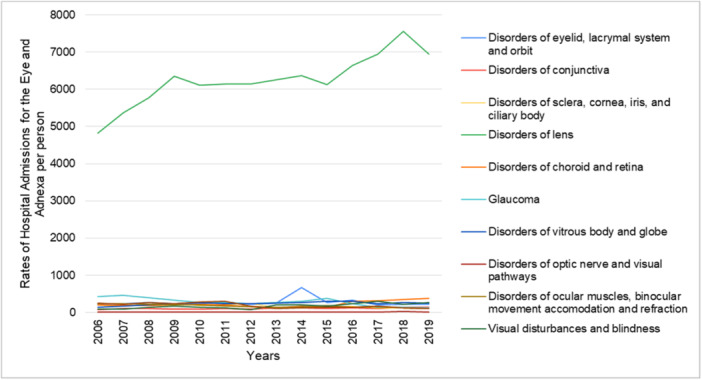
Hospital admission rates in Lebanon stratified by disease type between 2006 and 2019.

From 2006 to 2019, the highest increase in hospital admission rate was observed in visual disturbances and blindness, rising from 94 to 272 cases (1.99 to 4.7 per 100,000, *p* < 0.001). This was followed by disorders of the optic nerve and visual pathways, which increased from 6 to 15 cases (0.13 to 0.26 per 100,000, *p* > 0.05), and disorders of the conjunctiva, which rose from 77 to 146 cases (1.63 to 2.53 per 100,000, *p* < 0.01), representing increases of 136%, 104%, and 54%, respectively. Conversely, hospital admission rates for glaucoma and other disorders of the eye and adnexa decreased by 75.7% (from 424 to 126 per 100,000, *p* < 0.001) and 67.4% (from 248 to 99 per 100,000, *p* < 0.001), respectively (Figure 1 and Table 2).

**Figure 2:**
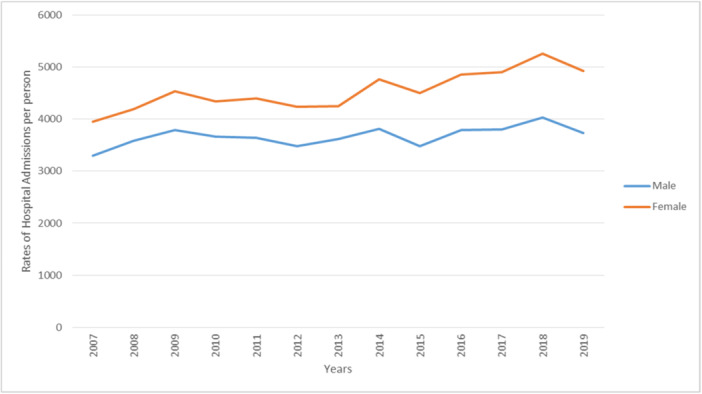
Overall rates of hospital admission stratified by gender.

**Figure 3:**
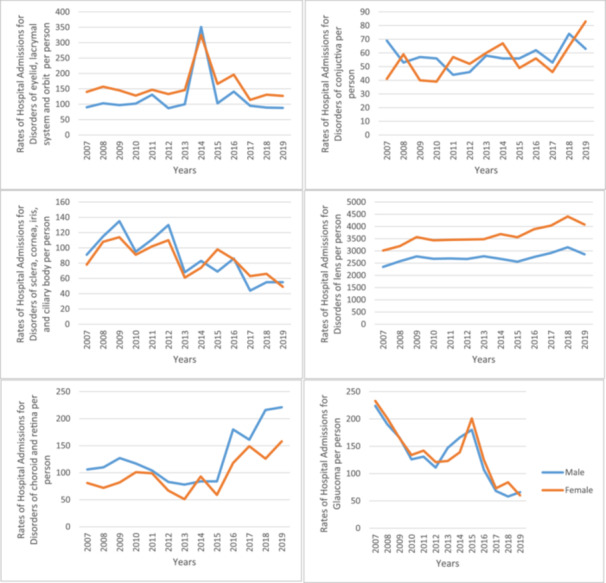
Overall rates of hospital admission stratified by disease type and gender (H00–H42).

**Figure 4:**
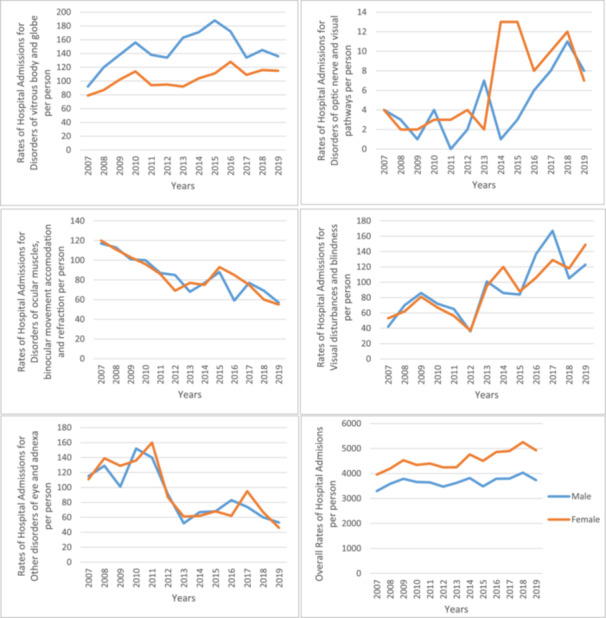
Overall rates of hospital admission stratified by disease type and gender (H43–H59).

The overall hospital admission rates for eye and adnexa‐related diseases were higher among females compared to males throughout the study period (Figure 2). However, the majority of admissions stratified by disease type were roughly equal between females and males. Hospital admissions for disorders of the eyelid, lacrimal system and orbit, disorders of the lens, and disorders of the optic nerve and visual pathways, were more common among females. In contrast, disorders of the choroid and retina, and disorders of the vitreous body and globe, were more common among males (Figures 3 and 4).

## 4 Discussion

To our knowledge, this is the first study to investigate trends in hospital admissions for eye and adnexa‐related diseases in Lebanon. Trends were assessed using a pre‐specified chi‐square χ² test for trend, in accordance with SAMPL recommendations. Our analysis demonstrates a highly significant increase in the hospital admission rate, rising by 5.9% from 6,675 (141.42 per 100,000) in 2006 to 8,660 (149.78 per 100,000) in 2019. This rise in admission rates may be associated with the growing population and increased life expectancy. Additional contributing factors likely include heightened health awareness, improved access to diagnostic technologies, and the implementation of screening programs. Notably, the overall hospital admission rate in Lebanon increased from approximately 214,000 in 2006 to about 320,000 in 2019, representing a 49% increase [19].

### 4.1 Overall Rates of Eye Hospital Admissions Stratified by Gender

The prevalence of eye and adnexa‐related diseases was higher among females than males throughout the study period (Figure 2). Similar findings have been demonstrated in a recent study assessing hospital admission rates for eye‐related problems in England and Wales [2]. Additionally, the female population in Lebanon has historically been larger than the male population, and women have a higher life expectancy [14]. These demographic factors may explain the higher number of eye‐related hospital admissions among females during this period. Furthermore, fluctuations in female sex hormones due to pregnancy, menstrual cycles, and menopause can trigger or even exacerbate various eye disorders, as sex hormone receptors are present in ocular tissues [19].

### 4.2 Rates of Eye Hospital Admissions Stratified by Disease Type

The most prevalent causes of hospital admissions for eye and adnexa‐related problems were disorders of the lens, with 87,548 cases (77.3%), followed by glaucoma and disorders of the eyelid, lacrimal system, and orbit, with 3,985 cases (3.4%) (Table 1). This pattern reflects the substantial prevalence of these disorders in Lebanon. Cataract is the leading cause of vision impairment and blindness in low‐income countries such as Lebanon, accounting for approximately half of all blindness cases, compared to less than 20% in high‐income countries [20]. Age is a significant risk factor for cataracts, and the WHO predicts an increase in acquired vision impairment due to global population growth and aging [1]. Nevertheless, the wider availability of surgical interventions for cataracts in these countries has led to a substantial rise in cataract surgeries over recent decades, reducing cataract‐related vision impairment and blindness [21]. Conversely, glaucoma is the second leading cause of blindness after cataracts and represents the primary cause of irreversible blindness. Primary open‐angle glaucoma is the most common type, with an estimated global prevalence of 68.56 million worldwide [22], projected to rise to 111.8 million by 2040 [23].

Disorders of the eyelid, lacrimal system, and orbit are frequent causes of hospital admissions [2]. As previously noted, the increase in admissions may be attributed to the aging population, which is at higher risk of developing these conditions. In addition, thyroid eye disease, often associated with hyperthyroidism, commonly presents with proptosis and eyelid retraction [24]. In the Middle East, the prevalence of thyroid diseases is estimated to range between 6% and 47% [25]. Early diagnosis and intervention, along with control of risk factors, may help reduce the risk of thyroid eye diseases [26]. Notably, smoking has been identified as a significant risk factor for Graves' ophthalmopathy [27]. The WHO estimates that smoking prevalence continues to rise in the Mediterranean Region, with approximately 35% of individuals aged 15 and older in Lebanon being smokers [28].

Our study shows that the most significant increase in hospital admissions for eye and adnexa‐related problems was attributed to visual disturbances and blindness (Table 2). Consistent with our findings, epidemiological studies worldwide have shown a substantial increase in the global prevalence of visual impairment and blindness by 91.46% between 1990 and 2019 [29]. This rise is associated with the growing number of individuals affected by cataracts, diabetic retinopathy, glaucoma, and age‐related macular degeneration. Socioeconomic factors play a crucial role in determining the burden of eye diseases, with visual impairment being more prevalent in low‐income countries [30]. A cross‐sectional study conducted in the United States found that individuals with lower levels of education and income were less likely to have had an eye care visit in the previous 12 months [31]. Additionally, low socioeconomic status has been associated with a higher prevalence of diabetic retinopathy among patients with diabetes [32]. Other factors affecting access to vision care include physical disabilities, cognitive dysfunction, and proximity to eye care centers [33]. These findings underscore the importance of implementing strategies to improve access to vision care services in Lebanon.

The second highest increase in hospital admissions for eye and adnexa‐related problems was attributed to disorders of the optic nerve and visual pathway, although this was not statistically significant (Table 2). This trend may indicate a heightened susceptibility of the population to optic nerve disorders, such as ischemic optic neuropathy, with age remaining a major risk factor [34]. Additionally, optic neuritis is frequently observed in patients with multiple sclerosis. Recent reports indicate a rising prevalence of multiple sclerosis in the Middle East, increasing from 5 to 30 per 100,000 population in the 1980s and 1990s to 65–85 per 100,000 in the 2020s [35].

### 4.3 Global Context

In recent years, the global burden of eye diseases and vision impairment has continued to grow, driven by population aging, increasing prevalence of chronic conditions such as diabetes, and persistent gaps in access to quality eye care services, especially in low‐ and middle‐income countries. According to the World Health Organization, at least 2.2 billion people worldwide have a vision impairment or blindness, and approximately 1 billion of these cases remain preventable or untreated, highlighting the ongoing global demand for effective eye care strategies and health‐system planning [1].

Trends in hospital admissions due to eye and adnexa disorders have likewise been documented in other national settings, with studies in the UK and Australia demonstrating significant increases in hospitalization rates over the past two decades, reflecting changing disease patterns, improved diagnostic capabilities, and greater health‐seeking behavior [2, 9].

Against this backdrop, our nationwide analysis of eye and adnexa‐related hospital admissions in Lebanon from 2006 to 2019 provides important and timely epidemiological evidence that can inform policymakers, health‐care planners, and clinicians about evolving care needs, help optimize resource allocation, and guide the development of targeted preventive and treatment strategies in a region where such comprehensive long‐term data have been lacking.

### 4.4 Potential Confounding and Interpretation of Trends

Given the retrospective and epidemiological nature of the MOPH database, our findings may be subject to residual confounding [36]. Temporal changes in hospital admission rates may be influenced not only by true variations in disease burden, but also by shifts in population age structure, sex distribution, health‐seeking behavior, access to ophthalmologic services, referral patterns, and improvements in diagnostic and surgical capacity over time. In particular, population aging, the increasing number of practicing ophthalmologists, and expanded availability of cataract and vitreoretinal surgery may partly account for the observed rise in lens‐related admissions and visual impairment‐related hospitalizations, while changes in outpatient management and screening practices could explain the decline in glaucoma and other categories. Therefore, the observed associations should be interpreted as epidemiological trends in hospital utilization rather than direct measures of disease incidence or causal effects. Nevertheless, the use of standardized ICD‐10 coding, population‐adjusted rates, and a unified national payer database over a 14‐year period provides robust and internally consistent estimates that are valuable for health system planning and hypothesis generation.

## 5 Limitations

The distribution of males and females for the year 2006 is unavailable. Data pertaining to admissions at private hospitals or those beyond the scope of coverage by the Ministry of Public Health (MOPH) are not included. Consequently, the dataset does not fully represent the entirety of the Lebanese population. Additional limitations of this study include the inability to account for potential confounding factors.

## 6 Conclusion

To our knowledge, this is the first nationwide epidemiological study to address eye and adnexa‐related hospital admissions in Lebanon. A significant increase in admission rates was observed between 2006 and 2019, with a clear female predominance. The rising trend may be attributed to various factors, including population growth, increased life expectancy, and underlying socio‐economic factors. Notably, the largest increases were seen in hospitalizations for visual disturbances, blindness, and disorders affecting the optic nerve and visual pathways. These findings underscore the need for strengthened preventive strategies and equitable access to eye care services, as well as continued national surveillance to guide health policy, resource allocation, and future research on eye and adnexal diseases in Lebanon and worldwide.

## Author Contributions

D.A., R.I., M.A., M.B., M.M., and J.B. conceptualized the study and drafted the manuscript. D.A., M.A., M.B., and M.M. collected the data. D.A., R.I., M.A., M.B., M.M., and J.B. analyzed data and drafted the manuscript. All authors have read and approved the final version of the manuscript. All authors have full access to all of the data in this study. The first and corresponding author, Dany Akiki, takes complete responsibility for the integrity of the data and the accuracy of the data analysis.

## Funding

The authors have nothing to report.

## Conflicts of Interest

The authors declare no conflicts of interest.

## Transparency Statement

The lead author Dany Akiki affirms that this manuscript is an honest, accurate, and transparent account of the study being reported; that no important aspects of the study have been omitted; and that any discrepancies from the study as planned (and, if relevant, registered) have been explained.

## Data Availability

The data supporting the findings of this study are publicly available from the Statistical Bulletin tables on the official website of the MOPH [9].
